# Supplementary material for: Getting More Out of Biomedical Documents with GATE's Full Lifecycle Open Source Text Analytics
Source: PLoS Comput Biol. 2013 Feb 7;9(2):e1002854. doi: 10.1371/journal.pcbi.1002854 (PMC3567135; doi:10.1371/journal.pcbi.1002854)
Supplement: Dataset S1 — GATE software. Dataset S1 bundled with this paper contains a distribution of GATE (or see http://gate.ac.uk/download/). (TGZ) [file pcbi.1002854.s001.tgz › plos-gate/doc/9MonthReport.html]

GATE2 9-month report


## GATE 2: 9-month report

### Hamish Cunningham, Kalina Bontcheva, Valentin Tablan, Yorick Wilks, 31st March, 2000

This is the first in a series of progress reports for the GATE 2 project
(EPSRC grant GR/M31699, running from July 1999 to summer 2002).
Our starting points are the original
GATE project (GR/K25267 - available at http://www.dcs.shef.ac.uk/nlp/gate/),
and the proposal document for the new project.

In sum, our aim is to relieve language processing R&D
workers from as much of the burden of routine software engineering as
possible. The original GATE project can claim to have achieved this goal
within the limited arena of certain types of language analysis program.
The new project seeks to reach a wider audience, and to move GATE onto the
Web using Java technology.

Our work over the last 9 months has covered 4 areas:

1. the development of several initial prototypes,
   linking up with the AVENTINUS, EUDICO and LOTTIE projects
   (you can see some of the stuff we did on
   our demos page);- a comprehensive requirements analysis for GATE 2, available from the
     publications page;- a start on developing the new version, directed top-down by the
       requirements analysis and bottom-up by the prototype code base (initial
       releases are available in Sheffield on
       the internal server);- setting up the new
         gate.ac.uk website
         for distribution of the system, and net-based
         access to NLP resources within GATE.
